# Programmed Cell Death 1 (PD-1) and Cytotoxic T Lymphocyte-Associated Antigen 4 (CTLA-4) in Viral Hepatitis

**DOI:** 10.3390/ijms18071517

**Published:** 2017-07-13

**Authors:** Hyosun Cho, Hyojeung Kang, Hwan Hee Lee, Chang Wook Kim

**Affiliations:** 1 College of Pharmacy, Duksung Women’s University, Seoul 01369, Korea; Hyosun1102@duksung.ac.kr (H.C.); leedh010700@duksung.ac.kr (H.H.L.); 2 Innovative Drug Center, Duksung Women’s University, Seoul 01369, Korea; 3 College of Pharmacy, Research Institute of Pharmaceutical Sciences and Institute for Microorganisms, Kyungpook National University, Daegu 41566, Korea; hkang72@knu.ac.kr; 4 Department of Internal Medicine, College of Medicine, The Catholic University of Korea, Seoul 06591, Korea

**Keywords:** programmed cell death 1 (PD-1), cytotoxic T lymphocyte-associated antigen 4 (CTLA-4), hepatitis A virus (HAV), hepatitis B virus (HBV), hepatitis C virus (HCV)

## Abstract

Virus-specific cluster of differentiation 8 (CD8+) cytotoxic T cells (CTL) recognize viral antigens presented on major histocompatibility complex (MHC) class I chains on infected hepatocytes, with help from CD4+ T cells. However, this CTL response is frequently weak or undetectable in patients with chronic hepatitis B virus (HBV) and hepatitis C virus (HCV) infection. Programmed cell death 1 (PD-1) and cytotoxic T lymphocyte-associated antigen 4 (CTLA-4) are receptors in the CD28 family of costimulatory molecules, providing inhibitory signals to T cells. The overexpressions of PD-1 and CTLA-4 in patients with viral infection have been shown to associate with functional impairment of virus-specific T cells. In acute viral hepatitis, PD-1 and CTLA-4 are up-regulated during the symptomatic phase, and then down-regulated after recovery. These findings suggest that PD-1 and CTLA-4 have protective effects as inhibitory molecules to suppress cytotoxic T cells which induce harmful destruction of viral infected hepatocytes in self-limited viral hepatitis. In chronic viral hepatitis, the extended upregulations of PD-1 and CTLA-4 are associated with T cell exhaustion and persistent viral infection, suggesting positive correlations between expression of immune inhibitory factors and the chronicity of viral disease. In this review, we summarize recent literature relating to PD-1, CTLA-4, and other inhibitory receptors in antigen-specific T cell exhaustion in viral hepatitis, including hepatitis A, B, C, and others.

## 1. Introduction

Programmed cell death 1 (PD-1) and cytotoxic T lymphocyte-associated antigen 4 (CTLA-4) are major inhibitory molecules that reduce T cell activation. A number of studies have reported overexpression of PD-1 and CTLA-4 on antigen-specific cluster of differentiation 4 (CD4+) or CD8+ T cells during chronic viral infections, including those by hepatitis B virus (HBV), hepatitis C virus (HCV), human immunodeficiency virus (HIV), and lymphocytic choriomeningitis virus (LCMV). The upregulation of PD-1, CTLA-4 and other inhibitory receptors have shown to associate with anti-viral effector T cell dysfunction. In viral hepatitis, HBV and HCV persist with functionally-impaired, virus-specific peripheral T cells as well as intrahepatic T cells that over-express PD-1 and CTLA-4. The functional restoration of virus-specific T cells by ex vivo or in vivo blocking PD-1 or CTLA-4 has been repeatedly confirmed. In this study, we review the current understanding of immune inhibitory receptors such as PD-1, CTLA-4, CD244, Lymphocyte activation gene 3 (*LAG-3*), T cell immunoglobulin domain and mucin domain-3 (Tim-3), and CD160 (expressed on virus-specific T cells in hepatitis).

## 2. T Cell-Mediated Anti-Viral Responses in Viral Hepatitis

The host immune defense against viral hepatitis depends on the interaction between innate and adaptive immune responses. The latter is essential in the successful control of viral infection [1]. T cells have important antiviral activities in patients with viral hepatitis caused by hepatitis A virus (HAV), HBV, HCV, and other hepatitis-related viruses. Virus-specific T cells kill virus-infected cells and activate B cells. The antiviral effect of T cells is affected by immune-stimulatory or immune-inhibitory molecules that are expressed on their surface. CD28 provides co-stimulatory signals that are essential for T cell activation and survival. CD28 is the receptor for CD80 (B7-1) and CD86 (B7-2), which are expressed on the surface of antigen presenting cells (APCs). PD-1 and CTLA-4 belong to the CD28 family of costimulatory molecules, but deliver inhibitory signals to T cells [2]. PD-1 binds to programmed cell death 1 ligand 1 (PD-L1) or PD-L2, which are expressed on the surface of APCs, and CTLA-4 binds to B7-1 (or B7-2) competitively with CD28 [2]. The chronicity of viral hepatitis is associated with the persistent expression of PD-1 and CTLA-4 [3,4].

### 2.1. Hepatitis A Virus (HAV)

Infection of the liver with HAV causes an acute self-limited disease that does not result in a chronic infection, unlike HBV or HCV. HAV-specific CD8+ T cell responses, including cytotoxicity and interferon γ (IFN-γ) production, were first reported in the blood of HAV infected patients [5]. CD8+ and CD4+ clones from liver biopsies of patients with acute hepatitis A were strongly cytotoxic, and released IFN-γ, which was correlated with the anti-viral response [6]. In HAV-infected chimpanzees, CD4+ T helper cells with activity against HAV provide earlier and better anti-viral activity than CD8+ T cells [7]. In this chimpanzee model, the frequency of HAV-specific CD4+ T cells targeting class II epitopes was much higher than that of CD8+ T cells, which is also closely linked with the control of viremia [7]. Recently, Choi et al. reported that the severity of liver injury is associated with decreased frequency of regulatory T cells (Treg) in blood from acute hepatitis A patients, which suggests that effector cells that contribute to immunopathology exist [8].

### 2.2. Hepatitis B Virus (HBV)

The natural disease progression of HBV infection is divided into four different phases according to the host adaptive immune responses. These are the immune tolerance, immune clearance, inactive, and reactivation phases of chronic hepatitis B [9]. HBV viral proteins play a prominent role in the process of viral antigen presentation and recognition by T cells. Hepatitis B virus core antigen (HBcAg) stimulates an efficient CD8+ T cell response that induces cytotoxicity of the infected hepatocytes [10]. Hepatitis B surface antigen (HBsAg), a major HBV protein, induces the T helper (Th)2-response [11]. Hepatitis B e antigen (HBeAg) induces the production of Thl cytokines such as interleukin (IL)-2 and IFN-γ by activation of CD4+ T cells [12]. Both HBV-specific CD8+ and CD4+ T cell responses are known to play a significant role in the control of HBV infection and liver inflammation. Enriched HBV-specific CD8+ T cells were found in the HBV-infected liver without hepatic immunopathology, and the inhibition of HBV replication is correlated with the increased abundance of circulating CD8+ T cells [13]. Thimme et al. reported that CD8+ cells are primarily responsible for viral clearance and disease pathogenesis during acute HBV infection in chimpanzees [14]. Interestingly, several groups have found that the peak CD8+ T cell production is frequently related with the greatest liver damage, even though the absolute quantity of HBV-specific CD8+ T cells is very low [15,16], which suggests that nonspecific bystander CD8+ T cell activation could contribute to liver inflammation [17]. In addition, the HBV-specific CD8+ T cell responses depend on the presence of CD4+ T cells in NOD/Scid mice transfected with a plasmid containing a replication-competent copy of the HBV genome, which indicates that CD4+ T cells act as a key regulator in the adaptive immune response to HBV infection [18]. In fact, patients with chronic HBV infection exhibit a weak virus-specific T cell response [19].

### 2.3. Hepatitis C Virus (HCV)

HCV is also a highly persistent pathogen that causes chronic liver inflammation, which later progresses to liver cirrhosis and hepatocellular carcinoma (HCC) [20,21]. HCV viral RNA and proteins actively participate in the host immune recognition of HCV. Initially, HCV activates TLR3 through RNA-dependent protein kinase R (PKR) and retinoic acid-inducible gene I (*RIG-I*) signals. Subsequently, signals are transmitted via mitochondrial antiviral signaling protein (MAVS), inducing IFNs and the Toll interleukin 1 (Toll-IL-1) receptor-domain-containing, adaptor-inducing IFN-β (TRIF). In fact, HCV NS3/4A protease can efficiently inactivate MAVS and TRIF, blocking IFN-β and interferon-stimulated genes (*ISG*) induction [22,23]. Walker et al. reported that HCV-specific memory CD8+ T cells are essential for long-term protection from chronic hepatitis C in chimpanzee models, and that the depletion of CD4+ T cells also resulted in persistent viremia, even though the functional memory CD8+ T cell response was maintained [24,25]. Both studies suggest that HCV-specific T cells are important to the resolution of HCV infection. Patients with chronic HCV infection showed functional changes in HCV-specific CD4+ T cells, including loss of both IFN-γ production and T cell proliferation [26,27].

### 2.4. Hepatitis Delta Virus (HDV) and Hepatitis E Virus (HEV)

There are few studies elucidating the role of virus-specific T cells in the hepatitis delta virus (HDV) or the hepatitis E virus (HEV) infections. HDV causes the most severe hepatitis when it superinfects patients with HBV infection. The activation of HDV-specific CD4+ or CD8+ T cells clear HDV RNA [28]. In contrast, HDV-specific T cell responses were not detected in patients with persistent HDV infection [29]. HEV infection causes self-limiting acute hepatitis, but possibly causes severe hepatitis during pregnancy [30]. Recently, Brown et al. reported that robust HEV-specific T cell responses are generated during acute hepatitis [31].

## 3. T Cell-Associated Hepatic Immune Regulation

The liver is typically known as a non-immunological compartment in our body, which is essential for mainly detoxification. However, the liver is also an active immunological structure for the production of acute phase proteins (APRs) and a variety of cytokines, and is surrounded by various populations of immune cells [32,33].

Diverse populations of adaptive immune cells, including CD4+ or CD8+ T cells, are found in the human liver, and activated CD8+ T cells are particularly enriched in the liver [34]. Populations of B cells are also present in the human liver and the innate-like CD5+ B cells, as well as the B cell subpopulation, are known to expand during viral infection [35].

The healthy liver is uniquely immunologically tolerogenic compared to other organs in the human body [36]. Resident hepatic cells, including Kupffer cells (KCs), myeloid dendritic cells (DCs), and myeloid-derived suppressor cells (MDSCs), contribute to maintaining hepatic tolerance. Endotoxin-induced KCs secrete IL-10 and prostaglandins, which downregulate the expression of co-stimulatory molecules on APCs, subsequently inhibiting stimulation of CD4+ T cells [37,38]. MDSCs also suppress hepatic immune responses through the production of IL-10 and TGF-β, the representative immunosuppressive cytokines [39]. These fundamental hepatic tolerogenic mechanisms support the persistence of hepatotropic pathogens such as HBV and HCV.

## 4. The Role of PD-1 and CTLA-4 in T-Cell Regulation

**Figure 1:**
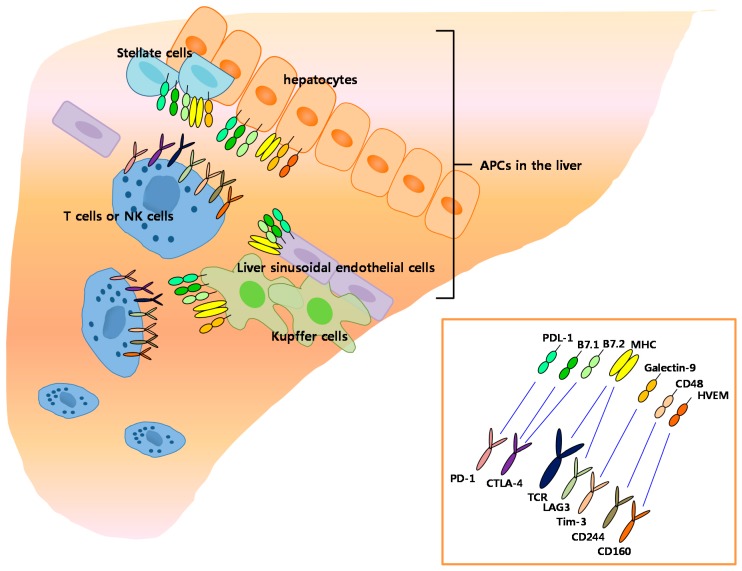
The interaction between immune inhibitory receptors and ligands in liver environment. programmed cell death 1 ligand 1, PD-L1; major histocompatibility complex, MHC; cluster of differentiation 48, 160, 244,CD48, 160, 244; CD80, 86, B7.1, 7.2; herpes virus entry mediator, HVEM; programmed cell death l, PD-1; cytotoxic T lymphocyte-associated antigen 4, CTLA-1; T-cell receptor, TCR; lymphocyte-activation gene 3, LAG3; and T cell immunoglobulin domain and mucin domain-3, Tim-3.

Recently, T cell-associated immune inhibitory molecules have been increasingly implicated as key players in the immunosuppression of chronic hepatic inflammation. The increased expression of programmed cell death protein (PD-1) on intrahepatic T cells is positively associated with T-cell exhaustion [40]. PD-1 binds to PD-L1 (or PD-L2), which is expressed on KCs, hepatocytes, hepatic stellate cells (HSCs), and liver sinusoidal endothelial cells (LSECs) [41,42,43]. These interactions between PD-1 and PD-L1 can induce liver immune tolerance via induction of T cell apoptosis or T cell dysfunction (Figure 1).

APCs in the liver express B7-1 (or B7-2), which specifically binds to CD28 on T cells. The interaction between B7-1 (or B7-2) and CD28 delivers co-stimulatory signals to antigen-primed T cells for a successful adaptive immune response. Interestingly, B7-1 (or B7-2) molecules can also ligate with CTLA-4 on T cells, which downregulates further T cell activation [40] (Figure 1).

Other negative T cell inhibitory molecules, including natural killer cell receptor CD244 [44], lymphocyte-activation gene 3 (*LAG-3*) [45], T cell immunoglobulin domain and mucin domain-3 (Tim-3) [46], CD160 [47], and adenosine A3 receptor (A3AR) [48] are related to T cell dysfunction in chronic hepatitis and HCC (Figure 1). Upregulation of CD244 was observed in virus-specific CD8+ T cells from chronic HBV and HCV infection [49,50]. Expression of LAG-3 is correlated with impaired effector function in HBV-specific CD8+ T cells in HCC patients [45]. The upregulation of Tim-3 is also associated with T cell exhaustion in chronic HBV infection [46]. CD160 is another inhibitory molecule linked with T cell exhaustion, and overexpression of CD160 was observed on HCV-specific CD8+ T cells from patients with chronic hepatitis C [50]. The A3AR was found to be overexpressed in the tumor and in the peripheral blood mononuclear cells of patients with HCC [48]. Collectively, these T cell–associated immune inhibitory molecules contribute to immunosuppression of hepatic inflammation, allowing chronic hepatitis or HCC progression.

### 4.1. PD-1

PD-1 is a 55 kDa glycoprotein that belongs to the CD28 immunoglobulin superfamily of transmembrane proteins [51]. The structure of PD-1 has 23% homology with CTLA-4; however, PD-1 acts as monomer and CTLA-4 is a disulfide-linked homodimer [51]. The cytoplasmic region of PD-1 has an immunoreceptor tyrosine-based inhibitory motif (ITIM) and an immunoreceptor tyrosine-based switch motif (ITSM) [52,53]. After binding of PD-L1 or PD-L2, PD-1 recruits the src homology 2 domain-containing tyrosine phosphatase 2 (SHP-2) to its ITSM domain and initiates the recruitment of tyrosine phosphatases, which produce an inhibitory signal that blocks the effects of the PI3K/Akt signaling pathway, leading to cell cycle arrest at G0/G1 phase and the induction of cell death through the down regulation of anti-apoptotic protein Bcl-xL [54,55]. Therefore, the activation of PD-1 facilitates the inhibition of both CD4+ and CD8+ T cell proliferation, which is associated with cell apoptosis and the inhibition of IL-2 secretion. Signaling through the PD-1 pathway affects the T cell response at the later effector stage, because upregulated expression of PD-1 was observed after persistent antigen exposure. Also, CD8+ T cells turn out to be more susceptible to regulation by PD-1: PD-L interactions, because they produce a low level of IL-2 [56].

PD-1 is expressed on resting and activated T cells, B cells, monocytes, and non-lymphoid cells, including those in the pancreas, placenta, and heart [57]. The ligands of PD-1, PD-L1, and PD-L2 are members of the B7 co-stimulatory molecule family and are located in the extracellular region [58,59]. PD-L1 is expressed on the surface of APCs and non-lymphoid tissues, including the liver, while PD-L2 is expressed on DCs and macrophages [58,59].

The interaction between PD-1 and PD-L1 or PD-L2 promotes apoptosis and secretion of the immunosuppressive cytokine IL-10 [60]. IFN-α, IFN-γ, or viral infection are all capable of upregulating the expression of PD-L1 on hepatocytes [41]. PD-L1 is also expressed on a variety of cancer cells, including melanoma, leukemia, glioblastoma, gastric cancer cells, bladder cancer cells, HCC, and breast cancer cells [61,62,63,64], while PD-1 is found on tumor-infiltrating lymphocytes (TILs) [65]. In addition, PD-L1 could stimulate the development of regulatory T cells by decreasing phospho-Akt, mTOR, and ERK2, as well as up-regulating PTEN. As a result, PD-L1 could inhibit the activation of T cells through the formation and retention of induced Treg (iTreg) cells [66].

A PD-L1-deficient mouse model shows selective impairment of CD8+ T cell function with severe hepatocyte damage in autoimmune hepatitis [67], which suggests that PD-1 is critical in regulating the cellular immune response to avoid autoimmune disorders.

Several studies have reported a progressive link between T cell exhaustion and PD-1 up-regulation [3,42,58,68,69], which indicates that blocking of the PD-1 and PD-L1 pathway could provide an effective therapeutic approach to recover the effector function of T cells.

### 4.2. CTLA-4

Fife et al. proposed that CTLA-4 signals present at an early stage in the lymph node during initiation of an immune response, whereas PD-1 signals are present at a later stage (at the tissue sites) to block T cell activity [2]. In other words, CTLA-4 regulates the immune response at the time of T cell activation. The stimulation of T cells is triggered by an antigen presented on APCs in association with the major histocompatibility complex (MHC) and co-stimulatory binding of CD28 to B7 [70]. CD28 is expressed on the surface of CD4+ and CD8+ T cells, and is the primary costimulatory molecule in T cell activation. TCR engagement and CD28 ligation lead to successful T cell–mediated adaptive immune responses [71]. The CD28 signaling pathway is initiated by binding of two well-defined ligands presented on APCs. The two ligands, B7-1 (CD80) and B7-2 (CD86), were thought to be the only ligands for CD28 and CTLA-4. However, a recent study reported that B7-H2, the ligand of inducible costimulatory (ICOSL), is also a ligand for CD28 and CTLA-4 in humans [72].

CTLA-4 seems to exhibit two different pathways to inhibiting T cell activation. First, CTLA-4 inhibits the positive costimulatory signaling of CD28 by competitively binding to B7-1 and B7-2, because CTLA-4 has a higher affinity for B7-1 and B7-2 than CD28 [70]. Second, CTLA-4 directly inhibits TCR signaling by increasing T cell motility and dominating the TCR-induced stop signal, which results in reduced contact between T cells and APCs. These pathways both lead to decreased cytokine production and proliferation of T cells [73]. These two different mechanisms seem to contribute to distinct functional outcomes of CTLA-4, which are either the rapid inhibition of T cell activation or the induction of T cell exhaustion [74].

The fundamental function of CTLA-4 is the control of CD28 binding with its shared ligands, B7-1 and B7-2, to prevent autoimmunity and to turn off an over-activation of the immune response. CTLA-4-deficient mice rapidly progress to lymphoproliferative disease with lymphocytic infiltration and tissue destruction [75]. CTLA-4-deficient mice also accumulate T cell blasts, which infiltrate the tissue of the liver, heart, lung, and pancreas [76]. The severe phenotype of CTLA-4-deficient mice implies a vital role for CTLA-4 in down-regulating the activation of T cells and preserving immunological homeostasis.

## 5. The Role of PD-1 and CTLA-4 in Viral Hepatitis

### 5.1. PD-1 and CTLA-4 in HAV

Upregulation of PD-1 and CTLA-4 on viral specific T cells from chronic HBV, HCV, and HIV infection has been repeatedly observed, and the expression of these immune inhibitory receptors is positively correlated with the functional impairment of T cells. Therefore, the sustained expression of PD-1 and CTLA-4 is attributed to the chronicity of viral infection. However, the phenotypic characteristics of virus-specific T cells in HAV infection are not clearly identified yet. Schulte et al. reported that HAV-specific activated CD8+ T cells could be detected ex vivo, and displayed the activated phenotype (CD38hi CD127lo) during HAV infection [77]. Also, Perrella et al. found that there was no statistically significant difference in the frequency and functional phenotype of CD4+ CD25+ Tregs in blood from HAV-infected patients compared to that of healthy controls [78]. There are few studies to investigate the phenotypic changes in the expression of PD-1 and CTLA-4 in acute viral hepatitis. One of our previous studies investigated the levels of PD-1 and CTLA-4 expression in CD4+ or CD8+ T cells from patients infected with hepatitis A. We found that both CD4+ and CD8+ T cells from patients with hepatitis A infection had significant levels of PD-1 and CTLA-4 during the symptomatic phase compared to those from the healthy controls, which was the first report to present the highly upregulated expression of PD-1 and CTLA-4 in hepatitis A [79]. In the same study, we also investigated if T cells from patients with non-viral acute hepatitis present phenotypic changes in the expression of PD-1 or CTLA-4. Interestingly, neither PD-1 nor CTLA-4 showed remarkable differences in expression levels compared to that in healthy controls [79]. Therefore, we concluded that the expression of PD-1 and CTLA-4 is highly associated with not only the chronicity of disease, but also viral factors. Also, there was a strong correlation between the expression of PD-1 and CTLA-4 in HAV infection, which indicated that PD-1 and CTLA-4 may be simultaneously expressed on the surface of T cells [79]. The PD-1 and CTLA-4 expressions during the symptomatic phase of acute hepatitis A seems to be dramatically reduced when patients are in recovery [79]. These findings suggest that PD-1 and CTLA-4 have a protective effect as inhibitory molecules, and can suppress cytotoxic T cells which induce harmful destruction of viral infected hepatocytes in acute self-limited viral hepatitis (Table 1).

### 5.2. PD-1 and CTLA-4 in HBV

Recently, many studies have reported that patients with chronic HBV infection present a weak immune response by impairment of T cell function (Table 1). Several mechanisms seem to contribute to the dysfunction of HBV-specific T cells, including suppressive cytokine production (IL-10, TGF-β) and an increased number of Tregs and co-inhibitory receptors, such as PD-1, T cell immunoglobulin domain and mucin domain-3 (TIM-3), CTLA-4, and CD244 (2B4), which result in a progressive loss of T cell function [49,71,80,81,82].

HBV-specific CD8+ T cells from peripheral blood mononuclear cells (PBMCs) of chronic HBV-infected patients have been known to express highly upregulated PD-1 [83,84], which is shown to be associated with HBV-specific T cell dysfunction [85]. PD-1 expression is also positively associated with HBV viremia in chronic hepatitis B infection [86]. Notably, HBV-specific CD8+ T cells from patients with acute HBV infection also showed impaired T cell function with enhanced expression of PD-1, but the PD-1+ population of T cells acquired a CD127+ memory phenotype with a loss of CD38 and PD-1 expression later in the resolving state. These recovered HBV-specific T cells presented an enhanced proliferative capacity, which was clinically correlated with viral clearance, suggesting that dysfunction of HBV-specific T cells is linked with expression of PD-1 as well as the expression of other signals [16]. In fact, PD-1 is considered as the principal inhibitory receptor among all inhibitory receptors expressed on exhausted T cells, because blocking the PD-1 pathway led to the strongest increase in function of ex vivo HBV-specific CD8+ T cell responses compared to blocking other inhibitory receptors (CD244, Tim-3, CTLA-4, and BTLA) [104]. Blocking the PD-1/PD-L1 interaction in ex vivo samples from CHB patients recovered the proliferation capacity and cytokine production (IFN-γ and IL-2) of both CD8+ and CD4+ T cells, which suggests that the PD-1/PD-L1 pathway plays a critical role in exhausted T cells during chronic HBV infections [84]. Interestingly, Intrahepatic HBV-specific CD8+ T cells expressed higher levels of PD-1 than their peripheral counterparts, and ex vivo treatment of anti-PD-L1 had a stronger effect on intrahepatic cells than on peripheral T cells. These findings suggest that intrahepatic HBV-specific T cells have better functional recovery after the blocking of PD-1, and therefore anti-PD-L1 could be a good therapeutic candidate for chronic HBV infection [105].

Although the molecular mechanism involved in the upregulation of PD-1 is not clearly explored yet, a recent study of LCMV infection in vivo reported that T-bet directly suppressed the transcription of PD-1 and resulted in lower protein expression of PD-1. In the same study, persistent antigenic stimulation caused downregulation of T-bet, which led to more severe exhaustion of CD8+ T cells in chronic viral infection [87]. In addition, the pro-inflammatory cytokine IL-12 effectively recovered the functional capacity of exhausted HBV-specific CD8+ T cells, down-regulated the expression of PD-1, and increased the expression of T-bet in an ex vivo sample of chronic hepatitis B (CHB) patients. Of note, a combination of IL-12 with PD-1 blockade increased CD8+ T cell functionality, which provides a new approach to controlling persistent viral infections [106]. Another study also informed that highly expressed T-bet in virus-specific CD8+ T cells during acute HBV and HCV infection was associated with natural recovery, while the lack of T-bet was more characteristic of progressive chronic infection. IL-12 induced selective phosphorylation of signal transducer and activator of transcription 4 (STAT4) in T-bet+ virus specific CD8+ T cells [107].

However, the ex vivo blockade of the PD-1 didn’t seem to fully recover the function of the HBV-specific CD8+ T cells in CHB patients [84], which implicates additional mediators. CTLA-4 (CD152) is another inhibitory receptor that drives T cell exhaustion during chronic viral infections [71], and ex vivo blockade of CTLA-4 has shown to provide a synergistic effect with blocking PD-1 in recovery of T cell exhaustion in HCV infection [40].

As mentioned earlier, successful T cell activation requires a T cell receptor-mediated signal, as well as a costimulatory signal by CD28. CTLA-4 is initially induced upon T cell activation; however, it inhibits CD28-dependent T cell activation by competitively binding to B7, the counterpart of CD28 [2].

The role of CTLA-4 in the pathogenesis of HBV infection was investigated at the genetic levels. Particular single nucleotide polymorphisms (SLP) of CTLA-4 were highly associated with HBV persistence, as well as HBV-specific T cell responses [88]. CTLA-4 commitment is reported to promote Th2 responses in HBV infection [90]. In chronic HBV infected patients, the highly expressed CTLA-4 in HBV-specific CD8+ T cells is positively correlated with HBV viral load, and the blockade of CTLA-4 increased the proliferation of IFN-γ-secreting HBV-specific CD8+ T cells, which suggests that modulating the expression of CTLA-4 can be another therapeutic approach in treating T cell exhaustion [89]. The underlying molecular mechanism for high CTLA-4 expression is associated with Bim-dependent cell apoptosis. The up-regulated expression of CTLA-4 in HBV-specific CD8+ T cells from CHB patients showed a positive correlation with the highest levels of Bim, and blocking CTLA-4 significantly decreased Bim expression [89]. Therefore, the rescue of T cell impairment using the blockage of CTLA-4 could be a practical strategy to eliminate HBV infection.

### 5.3. Other Inhibitory Receptors in HBV

There are several other receptors that are highly upregulated in the context of T cell impairment during chronic HBV infection (Table 1). CD244 is a transmembrane protein, which belongs to the immunoglobulin superfamily and is also expressed by natural killer cells and CD8+ T cells [44]. CD244 was first identified on exhausted CD8+ T cells in chronic LCMV infection [82]. HBV-specific CD8+ T cells expressed higher levels of CD244, both in the peripheral blood and liver, compared to that in the acute phase of infection in CHB patients [49]. The same study also reported that the blockade of CD244 recovered the function of HBV-specific CD8+ T cells, including proliferation, cytokine production, and cytotoxicity.

Lymphocyte activation gene 3 (*LAG-3*) was identified as a novel lymphocyte activation gene, found in activated T and NK cells [108]. Blocking PD-1 and LAG-3 concurrently showed a synergistic effect on improvement of T cell responses, which suggests that LAG-3 has an inhibitory role in CD8+ T cell exhaustion in chronic viral infections [82]. Kennedy et al. addressed that the expression of LAG-3 on HBV-specific CD8+ T cells was up-regulated in progressive stages of CHB [91]. LAG-3 expression was also increased in tumor infiltrating HBV-specific CD8+ T cells obtained from HCC patients, which correlated with severe functional defects at the tumor site [45].

Tim-3 is another inhibitory molecule expressed on T cells, which stimulates the cell death of Th1 cells and induces peripheral tolerance [109]. In CHB patients, the increased expression of Tim-3 is involved in disease progression of CHB and contribute to skewing of the Th1/Tc1 response, leading to persistency of the HBV infection [92]. Tim-3 was highly upregulated on HBV-specific CD8+ T cells, which are functionally impaired and cannot secrete IFN-γ and tumor necrosis factor α (TNF-α). The blockade of Tim-3 resulted in the proliferation of HBV-specific CD8+ T cells. Interestingly, Blocking Tim-3 combined with PD-1 enhanced the antiviral responses in some CHB patients [46]. Ju et al. also addressed that a significant increase in Tim-3 expression in PBMCs, and abundance of circulating NK cells and liver-infiltrating lymphocytes (LILs) was obtained from CHB patients. Ex vivo blockade of Tim-3 increased the cytotoxicity of PBMCs or NK cells obtained from CHB patients [110]. Blocking Tim-3 signaling also increased the proliferation of tumor-infiltrating T cells in HBV-associated HCC patients, and the number of Tim-3+ tumor-infiltrating cells was negatively link with patients’ survival [111]. In addition, specific polymorphisms of the *Tim-3* gene in patients with CHB seem to be associated with viral persistency and HCC development [112].

### 5.4. PD-1 and CTLA-4 in HCV

Acute HCV infection can be recovered within a few months, but most HCV infections become chronic, and develop into liver fibrosis, liver cirrhosis, or HCC [20]. HCV-specific CD8+ T cells play a primary role in the control of viral infection in the acute phase [113]. HCV-specific CD8+ T cells had upregulated PD-1 expression during the acute stage of hepatitis C, but gradually expressed more CD127 in patients with resolving self-limited hepatitis C than in acute hepatitis B. In contrast, in patients with chronically evolving hepatitis C, CD127 expression continued to be negative with persistent PD-1 expression [114]. The effector function of HCV-specific CD8+ T cells becomes deeply impaired during chronic HCV infection, which results in persistent viral infection [115,116].

The upregulation of PD-1 may be one of the main mechanisms responsible for impairment of HCV-specific T cells during chronic HCV infection [93,94]. Although PD-1 is up-regulated on all HCV-specific CD8+ T cells during the early phase of HCV infection, its expression is modulated after the acute phase depending on the disease progression [117]. In the case of self-limited infection, HCV-specific CD8+ T cells have decreased PD-1 expression and obtain a CD127+ phenotype, which is an IL-7 receptor and plays a critical role in T cell survival [16].

HCV-specific CD8+ T cells with high levels of PD-1 were not capable of producing IFN-γ, TNF-α, IL-2, perforin, and granzyme B [95]. The expression of PD-1 on HCV-specific CD8+ T cells was also correlated with impaired proliferation capacity [3]. Interestingly, the level of PD-1 expression on intrahepatic HCV-specific CD8+ T cells from chronically infected patients was much higher than the level of PD-1 on circulating HCV-specific CD8+ T cells. These highly PD-1-positive intrahepatic CD8+ T cells were deeply dysfunctional, and their phenotype was considerably different from that of circulating CD8+ T cells in terms of increased CTLA-4, and reduced CD28 and CD127 expression [95].

The ex vivo blockade of PD-1 by anti-PD-L1 antibodies improved the function of HCV-specific CD8+ T cells, including proliferation and cytokine production of IFN-γ and IL-2 [3]. Jeong et al. reported that ex vivo blocking of PD-1 significantly increased the frequency of IFN-γ-producing HCV-specific CD4+ and CD8+ effector T cells and cytokine production such as IL-2. The production of perforin was also increased in HCV-specific CD8+ T cells [118]. Furthermore, restoration of HCV-specific T cell functions by the in vitro PD-1/PD-L1 blockade showed a synergistic effect with PEG-IFN-α treatment [119].

However, the ex vivo blockade of PD-1 was not sufficient to recover the function of intrahepatic HCV-specific CD8+ T cells, which were shown to have a much higher PD-1 expression. In fact, intrahepatic HCV-specific CD8+ T cells failed to proliferate and secrete IFN-γ and cytolytic molecules (perforin, CD107a) in the presence of anti-PD-L1 antibodies, which suggests the existence of other inhibitory molecules such as CTLA-4 in the liver [95]. Surprisingly, CTLA-4 was preferentially upregulated in intrahepatic PD-1+ T cells but not in circulating blood PD-1+ T cells in chronic HCV-infected patients [40]. The effector functions of PD-1/CTLA-4 co-expressed intrahepatic T cells were fully rescued by blocking both PD-1 and CTLA-4 ex vivo, but not blocking PD-1 or CTLA-4 alone, which suggests that both PD-1 and CTLA-4 contribute to HCV-specific T cell dysfunction in the liver [40].

As mentioned previously, many studies have emphasized the role of PD-1 signaling in the exhaustion of HCV-specific CD8+ T cells and how blocking PD-1 could recover the function of HCV-specific CD8+ T cells. In fact, several groups have investigated the possibility of the PD-1 blockade being combined with the use of a therapeutic vaccine, because therapeutic vaccines fail to induce a strong T cell response owing to their tolerogenic-like T cells [120,121]. Theoretically, this approach seems very optimistic if inhibitory molecules like PD-1 are blocked when the vaccine is administered with the aim of enhancing the specific immune response. Ha et al. addressed the possibility of combining the PD-1 blockade and vaccine in an LCMV mouse model. In that study, in vivo blocking PD-1/PD-L1, in combination with therapeutic vaccination, synergistically enhanced the functionality of the CD8+ T cell responses and improved LCMV control [121]. Even though these results are encouraging, blocking PD-1 pathways could contribute to the development of autoimmune disorders [122]. Thus, more research is required before blocking PD-1 is proper for anti-HCV reagents in chronic HCV infection.

The critical role of CTLA-4 in chronic HCV infection was first addressed by Yee et al., who found the associations of CTLA-4 polymorphisms with sustained response to IFN-α and ribavirin therapy for chronic HCV infection [96]. Nakamoto et al. discovered that CTLA-4 is highly upregulated in intrahepatic HCV-specific CD8+ T cells from chronically infected patients, especially on highly PD-1+ T cells. They identified that these PD-1+/CTLA-4+ CD8+ T cells were deeply dysfunctional HCV-specific cells and were not responsive to the ex vivo PD-1/PD-L blockade, unlike circulating HCV-specific CD8+ T cells, suggesting that active HCV-specific stimuli induces a profound functional exhaustion of T cells, especially in the liver [95]. Their subsequent study reported that severe effector dysfunction of intrahepatic CD8+ T cells caused by PD-1/CTLA-4 co-expression was synergistically reversed by the PD-1/CTLA-4 blockade, and this functional response to combined blockade was directly connected with CTLA-4 expression, and lost with CD28-depletion and CD4-independent [40]. The same functional recovery effect was observed in circulating PD-1/CTLA-4 coexpressed HCV-specific CD8+ T cells during acute hepatitis C infection [40].

### 5.5. Other Inhibitory Receptors in HCV

CD244 is upregulated in chronic hepatitis C (CHC) patients and the increased expression of CD244 is positively associated with the level of PD-1 expression. Importantly, this CD244+/PD-1+ phenotype was correlated with an impaired proliferative capacity and intermediate differentiation in HCV-specific T cells [97]. Schlaphoff et al. further demonstrated that enhanced expression of CD244 was observed on HCV-specific CD8+ T cells in both acute and chronic hepatitis C and there was a positive relationship between the level of CD244 and the impairment of HCV-specific CD8+ T cell function [50]. Similarly, the expression of CD244 was higher in HCV-specific intrahepatic CD8+ T cells than in T cells from blood in CHC patients [98]. This data shows that CD244 is another inhibitory molecule that could be modulated for the restoration of HCV-specific T cell functioning.

Increased expression of LAG-3 was found on intrahepatic T cells from chronic HCV infection compared with that in T cells from blood [98]. Interestingly, HCV-specific CD8+ T cells from patients with sustained virological responses expressed much higher levels of LAG-3, suggesting the existence of different populations of cells that even have differential expression of inhibitory makers [98]. Chen et al. addressed that the frequency of LAG-3 on intrahepatic and peripheral CD8+ T cells was higher in CHC patients, compared with that in healthy controls. The ex vivo blockade of LAG-3 reversed the function of HCV-specific CD8+ T cells in CHC patients with respect to cell proliferation, cytokine (IFN-γ, TNF-α, granzyme B, and perforin), expression, and cytotoxicity [99].

Tim-3 is another immune inhibitory receptor that restricts the activation of T cell responses [92]. HCV-specific CD8+ T cells were shown to express higher Tim-3 compared to EBV- and CMV-specific T cells, which were correlated with liver disease progress and viral load [100]. Increased expression of Tim-3 was also found on NK cells; however, the high level of Tim-3 was not recovered by IFN-α antiviral therapy [123]. The frequency of PD-1+ Tim-3+ HCV-specific CD8+ T cells is much less than that of PD-1- Tim-3- CD8+ T cells in patients with resolving infection, and Tim-3+ HCV-specific CD8+ T cells were especially enriched in the liver. Moreover, ex vivo blocking of Tim-3 improved the proliferation capacity of HCV-specific CD8+ T cells [101]. Blocking Tim-3 signaling using miR-155 enhanced IFN-γ production in NK cells ex vivo [124]. In addition, there is an association between Tim-3 polymorphisms and F protein with the outcomes of HCV infection [102]. Recently, the ex vivo Tim-3 blockade, in combination with TLR3 activation, induced an antiviral immune response in patients with chronic HCV infection [102], which suggests that the manipulation of Tim-3 expression could provide a rational target against chronic HCV infection.

The expression of CD160 was also reported to increase in HCV-specific CD8+ T cells obtained from CHC patients, and was highly related with T cell exhaustion [50,97,98]. Viganò et al. addressed that increased expression of CD160 on HCV-specific CD8+ T cells in CHC patients was not related with PD-1 expression. The ex vivo blocking CD160/CD160L interaction rescued the proliferation of HCV-specific CD8+ T cells [103].

## 6. Clinical Application of PD-1 and CTLA-4 for Viral Hepatitis

Immune inhibitory methods using anti-PD-1 or anti-CTLA-4 monoclonal antibodies have changed the controlling of patients with melanoma. Many PD-1 and CTLA-4 inhibitors, either as a single reagent, or in combination, have been permitted or tested in clinical trials by the United States Food and Drug Administration for the treatment of metastatic melanoma and other advanced cancers.

Nivolumab and pembrolizumab are recently developed PD-1 blocking reagents. Nivolumab is a human anti-PD-1 monoclonal immunoglobulin-G4, which blocks ligand binding. It was initially introduced as a promising anti-cancer candidate in phase I clinical trials with diverse tumors, including metastatic melanoma, non-small cell lung cancer, and renal cell cancer. Objective anti-tumor reponses were observed in one in four to one in five patients with 14% of grade 3 or 4 adverse events in advanced melanoma [125]. Subsequently, nivolumab was approved in phase 3 clinical trials for previously treated advanced non-small cell lung cancer and renal cell carcinoma [126,127]. Pembrolizumab was also approved in a phase 1 study for PD-L1+ advanced non-small cell lung cancer and in a phase 3 study for the treatment of advanced melanoma [128,129]. Ipilimumab and tremelimumab are monoclonal antibodies targeting CTLA-4 for cancer therapy. Ipilimumab treatment induced prolonged survival in phase II and phase III trials for advanced melanoma, and induced a plateau in the survival curve at nearly 3 years [130]. However, tremelimumab was unsuccessful at reaching numerical significance in a phase III trial for metastatic melanoma [131].

Among these drugs, only nivolumab and tremelimumab have completed clinical trials for viral hepatitis–related disease. Nivolumab was tested in 54 CHC patients, many of whom had undergone IFN-α treatment. Even though six patients showed immune-associated adverse events, including hyperthyroidism, nivolumab was well accepted in CHC patients [132]. The anti-tumor and anti-HCV effect of tremelimumab was also tested in 21 CHC patients with HCC. Tremelimumab exhibited a good safety profile without any severe immune-related adverse events. The disease control rate was about 76.4% and a significant drop in viral load was observed. The anti-viral effect was shown to correlate with an increased specific anti-HCV immune response [133].

The combination of an anti-PD-1 and an anti-CTLA-4 antibody was more effective than the individual monotherapies in advanced melanoma [134]. However, adverse drug responses (ADRs) were found to be more common in the combined treatment, and with more severe symptoms. The ADRs include toxicities in skin, gastro intestine, endocrine, kidney, and liver. Hepatic events seem very common under combination of nivolumab and ipilimumab, with about 17% grade 3–4 adverse events, compared to 2% with nivolumab and 0.1% with ipilimumab alone [135]. Therefore, the combined therapeutic approach with nivolumab and ipilimumab should be carefully considered, especially for the treatment of viral hepatitis such as HBV and HCV.

## 7. Conclusions

In summary, PD-1 and CTLA-4 are receptors in the CD28 family of costimulatory molecules, which induce T cell activation as well as delivering inhibitory signals to T cells. Viral hepatitis, including HAV, HBV, and HCV, was shown to have differential levels of PD-1 and CTLA-4 expression depending on disease progression. T cells obtained from HAV-infected patients had significantly increased expression of PD-1 and CTLA-4 during the symptomatic phase. Also, HBV or HCV-specific T cells obtained from chronically infected patients presented highly upregulated PD-1 and CTLA-4, as well as virus-specific T cell dysfunction. Interestingly, the level of PD-1 and CTLA-4 expression on intrahepatic T cells was much higher than that in circulating T cells, and is positively correlated with profoundly impaired function in intrahepatic T cells.

Blocking the PD-1 or CTLA-4 pathways is an interesting possible strategy to restore virus-specific T cell responses by modulating immune checkpoint molecules. These regulations resolve T cell exhaustion in chronic viral hepatitis and HCC.

## Figures and Tables

**Table 1:** Overexpressed inhibitory receptors in viral hepatitis.

Infection	Acute/Chronic	Overexpressed Inhibitory Receptors	Ligands	Significance	References
HAV	Acute	PD-1	PD-L1, PD-L2	Disease severity	[79]
		CTLA-4	B7-1, B7-2 B7-H2	Disease severity; increased ALT, AST	[79]
HBV	Chronic	PD-1	PD-L1, PD-L2	Chronicity	[83]
				T cell dysfunction	[84,85]
				Viral persistence	[86]
				Downregulation of T-bet	[87]
		CTLA-4	B7-1, B7-2	Chronicity, T cell dysfunction	[40]
				Viral persistence	[88,89]
				Th2 promotion	[90]
				Upregulation of Bim	[89]
				Polymorphism	[88]
		CD244	CD48	Chronicity	[49]
		LAG-3	MHC class II	Chronicity	[91]
				Hepatocellular carcinoma	[45]
		Tim3	Galectin-9	Chronicity	[92]
HCV	Chronic	PD-1	PD-L1, PD-L2	T cell dysfunction	[93,94]
				Viral persistence	[93]
				Highly positive in liver	[95]
		CTLA-4	B7-1, B7-2	T cell dysfunction	[95]
			B7-H2	Highly positive in liver polymorphism	[96]
		CD244	CD48	T cell dysfunction	[97]
				Highly positive in liver	[98]
		LAG-3	MHC class II	Highly positive in liver	[98]
				T cell dysfunction	[99]
		Tim-3	Galectin-9	Chronicity, persistent viremia	[100]
				Highly positive in liver	[101]
				Viral persistence polymorphism	[102]
		CD160	HVEM	Chronicity, T cell dysfunction	[103]
